# Development and validation of an integrated DNA walking strategy to detect GMO expressing cry genes

**DOI:** 10.1186/s12896-018-0446-x

**Published:** 2018-06-27

**Authors:** Marie-Alice Fraiture, Julie Vandamme, Philippe Herman, Nancy H. C. Roosens

**Affiliations:** 1 Scientific Institute of Public Health (WIV-ISP), Platform of Biotechnology and Bioinformatics (PBB) by: Sciensano, Transversal & Applied Genomics (TAG), J. Wytsmanstraat 14, 1050 Brussels, Belgium; 2 Scientific Institute of Public Health (WIV-ISP), Operational Direction Expertise, Service provisions & Customer relations by: Sciensano, Scientific Direction Expertise, Service provisions & Customer relations, J. Wytsmanstraat 14, 1050 Brussels, Belgium

**Keywords:** GMO, Detection, qPCR, Characterization, Identification, DNA walking

## Abstract

**Background:**

Recently, an integrated DNA walking strategy has been proposed to prove the presence of GMO via the characterisation of sequences of interest, including their transgene flanking regions and the unnatural associations of elements in their transgenic cassettes. To this end, the p35S, tNOS and t35S pCAMBIA elements have been selected as key targets, allowing the coverage of most of GMO, EU authorized or not. In the present study, a bidirectional DNA walking method anchored on the CryAb/c genes is proposed with the aim to cover additional GMO and additional sequences of interest.

**Results:**

The performance of the proposed bidirectional DNA walking method anchored on the CryAb/c genes has been evaluated in a first time for its feasibility using several GM events possessing these CryAb/c genes. Afterwards, its sensitivity has been investigated through low concentrations of targets (as low as 20 HGE). In addition, to illustrate its applicability, the entire workflow has been tested on a sample mimicking food/feed matrices analysed in GMO routine analysis.

**Conclusion:**

Given the successful assessment of its performance, the present bidirectional DNA walking method anchored on the CryAb/c genes can easily be implemented in GMO routine analysis by the enforcement laboratories and allows completing the entire DNA walking strategy in targeting an additional transgenic element frequently found in GMO.

**Electronic supplementary material:**

The online version of this article (10.1186/s12896-018-0446-x) contains supplementary material, which is available to authorized users.

## Background

With the aim to respect the consumer’s freedom of choice as well as to guarantee the traceability in the food and feed chain, the presence of genetically modified organisms (GMO) is controlled by enforcement laboratories in many countries. To this end, three main successive steps, namely the screening, the identification and the quantification, are commonly performed using the real-time PCR technology (qPCR). More precisely, a set of markers targeting transgenic elements commonly found in GMO (i.e., p35S and tNOS) or not is tested in a single qPCR screening analysis allowing the detection of potential GMO. This approach covers a large spectrum of GMO and discriminates certain GM events. According to qPCR observed signals, a list of the GM events that are potentially present in the sample is drawn up. The corresponding event-specific methods are then applied and the identified GM events are subsequently quantified [1, 2]. However, even if this GMO routine analysis is perfectly suitable for EU authorized GMO, its application to EU unauthorized GMO implies two main issues. On the one hand, the elements targeted during the screening step are originating from natural organisms, such as the promoter p35S from the Cauliflower Mosaic Virus (CaMV) and the terminator tNOS from *Agrobacterium tumefaciens*. Consequently, if the signals observed during the qPCR screening could not be explained by the presence of EU authorized GMO, the presence of EU unauthorized GMO could only be suspected because the qPCR screening data could also be due to the presence of naturally occurring microorganisms in the sample. On the other hand, with a sample composed of EU authorized and unauthorized GMO possessing both the same targeted transgenic element (i.e., p35S), the explanation of the qPCR screening signals by the identification of EU authorized GM events will conceal the presence of EU unauthorized GMO in the tested sample since no further analysis will be performed [3].

To avoid missing the presence of EU unauthorized GMO, the DNA walking approach has been investigated in order to characterize unknown sequences of interest surrounding known sequences [4–8]. In this context, we recently proposed an approach, integrated to the GMO routine analysis, allowing to indubitably prove by the sequence information the presence of unauthorized GMO. Following positive signals for key transgenic elements (p35S, tNOS and/or t35S pCAMBIA) in the qPCR screening analysis, a DNA walking assay, anchored on the detected key transgenic elements, is applied bi-directionally to obtain information about the sequences surrounding the known transgenic elements. In this way, the presence of EU unauthorized GMO is highlighted via the characterization of these sequences presenting their transgene flanking regions and/or unnatural associations of elements from their transgenic cassettes [9–12]. Regarding the choice of the key transgenic elements, the combination of p35S, tNOS and t35S pCAMBIA allows covering most of GMO. In addition, t35S pCAMBIA allows to target specifically some EU unauthorized GMO (~ 30%) since this transgenic element is not found in EU authorized GMO [9–12].

In order to strengthen this strategy regarding the characterization of EU unauthorized GMO (additional EU unauthorized GMO sequences as well as additional EU unauthorized GMO possessing none of the previously targeted key transgenic elements), we present here the development of a new bidirectional DNA walking method anchoring on the region targeted by the Cry1Ab/c qPCR screening marker, which targets synthetic Cry1Ab, Cry1Ac and Cry2Ab2 genes (namely here CryAb/c genes). These synthetic Cry genes have been used to produce several GMO to give them an insect resistance property through the Bt toxin production (*Bacillus thuringiensis* Cry protein). To date around 200 GM events of that kind have been already approved for commercial release in different countries since 20 years ago [13–15].

Using several GM events possessing the targeted elements (CryAb/c genes) in their transgenic cassette, the proposed bidirectional DNA walking method was first developed in term of feasibility. Second, this DNA walking method was evaluated for its sensitivity since GMO are often present in low amounts in the tested samples. Finally, its applicability in GMO routine analysis has been tested on a GeMMA Scheme Proficiency Test food matrix, mimicking a real-life sample, presenting a positive signal for the Cry1Ab/c marker in qPCR screening.

## Results

### In silico study

**Fig. 1:**
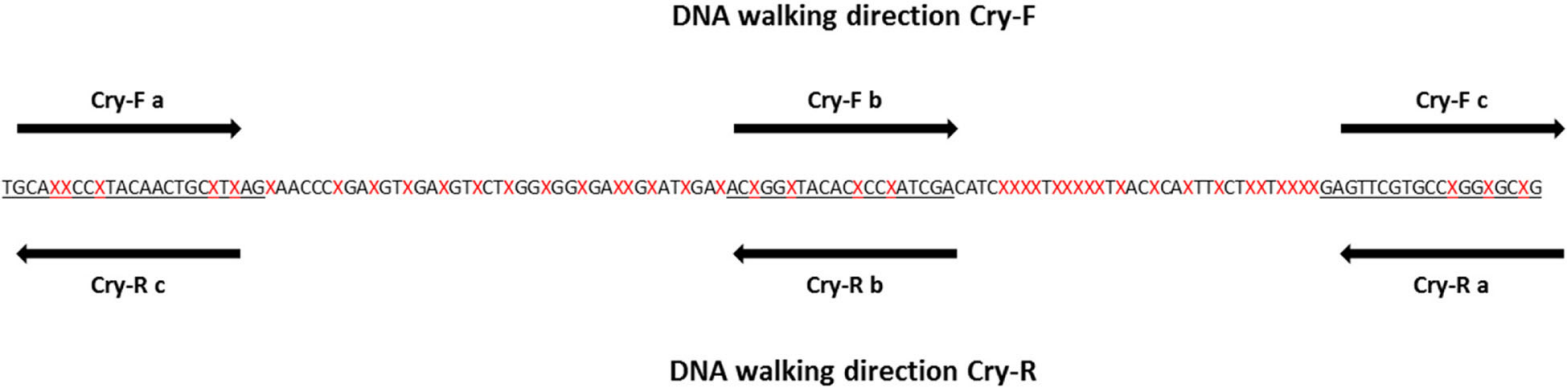
Annealing position of the target-specific primers. The primers used in the DNA walking Cry-F and Cry-R walking directions are respectively indicates above and below the consensus sequence. The walking direction is indicated by the arrows. The consensus sequence, based on the alignment of the targeted CryAb/c sequences from the Bt11, MON87701, MON87751, MON531, Bt176, T304–40, Bt63, MON810, KeFeng-6, MON89034, MON15985 and 281–24-236 × 3006–210-23 events, indicates the nucleotide variations between all the analysed GM events by the red crosses

As required by the DNA walking approach, three target-specific primers have to be designed on the CryAb/c genes. The couple of primers targeting the CryAb/c elements used for the qPCR screening step was selected. An additional target-specific primer was designed close at hand in taking into account the nucleotide variations of the targeted region between the different GM events positive to the qPCR Cry1Ab/c screening marker. In order to characterize as much as possible the regions surrounding the CryAb/c elements, two walking directions (Cry-F and Cry-R) were developed (Fig. 1; Table 1; Additional file 1) [13].

**Table 1:** Oligonucleotide primers used for the real-time PCR assays, the DNA walking methods and the PCR confirmation of the Cry sequence from the T304–40 event

Methods	Oligonucleotide names	Oligonucleotide sequences	Product sizes (bp)	References
SYBR®Green qPCR	Cry1Ab/c-F	ACCGGTTACACTCCCATCGA	73	[13]
	Cry1Ab/c-R	CAGCACCTGGCACGAACTC		[13]
DNA Walking	Cry-F a	TGCATTCCATACAACTGCTTGAG		This study
	Cry-F b (Cry1Ab/c-F)	ACCGGTTACACTCCCATCGA	/	[13]
	Cry-F c	GAGTTCGTGCCAGGTGCTG		[13]
DNA Walking	Cry-R a (Cry1Ab/c-R)	CAGCACCTGGCACGAACTC		[13]
	Cry-R b	TCGATGGGAGTGTAACCGGT	/	[13]
	Cry-R c	CTCAAGCAGTTGTATGGAATGCA		This study
PCR	T304–40-Cry-F	ACATGGATTATGGCCCACAT	787	This study
	T304–40-Cry-R	GTTGGCAGCTTGCACATAGA		This study

### Development the bidirectional DNA walking method anchored on the CryAb/c genes

The present bidirectional DNA walking method anchored on the CryAb/c genes has been developed and validated, using single GM events containing the CryAb/c genes, similarly to the previously described bidirectional DNA walking methods anchored on the t35S pCAMBIA, p35S and tNOS elements [9–12]. In order to initially assess their feasibility, the two walking directions of the DNA walking method anchored on the CryAb/c elements have been independently tested on 2000 HGE (Haploid Genome Equivalent; also referred as copy number) of several GM events possessing the targeted sequences (Table 2). For each walking direction, several amplicons were observed for all tested GM events, with a size range going approximately from 200 bp to more than 2000 Kbp. Given that each GM event was individually tested for each DNA walking direction, the generated amplicons were thus expected to result from the combination of one target-specific primer and four degenerated random tagging (DRT) primers. Consequently, these amplicons were expected to be amplified from the same region, with a similar starting point determined by the used target-specific primers, and may present variations in term of length due to the annealing sites of the DRT primers. Therefore, in order to simplify the analysis workflow, for each tested GM events, the longest and easiest selectable amplicon, which contained the most informative sequence, was sequenced using the Sanger technology for each walking direction to evaluate the specificity of the methods (Table 2, Additional file 2).

**Table 2:** Analysis of the bidirectional DNA walking method, including the Cry-F and Cry-R walking direction, applied on GM events (2000 HGEs) containing the CryAb/c genes, using the four different mixes of DRT primers (A-D)

	Cry-F				Cry-R			
Bt11 maize	**+**				**+**			
	**A**	**B**	**C**	**D**	**A**	**B**	**C**	**D**
	**550**	**500**	*650* *950* *1250*	*450* **1000** 1400* **1800**	400* *800*	*> 2000*	*500* *1000* *1400*	*550* *1300* *1600*
MON810 maize	**+**				**+**			
	**A**	**B**	**C**	**D**	**A**	**B**	**C**	**D**
	550* *850* *1300*	*650*		**400**	250*	*770*	*350* **500**	*260*
T304–40 cotton	**+**				**+**			
	**A**	**B**	**C**	**D**	**A**	**B**	**C**	**D**
	**550** *800*	*350*	*450* *500* *600* 800* *1200*	**400** *600*	*550*	*200*	*200* *450* *550*	*200* *450* 700*
MON531 cotton	**+**				**+**			
	**A**	**B**	**C**	**D**	**A**	**B**	**C**	**D**
	**550**	500*	*950*	*450* **1000** **1700** *1900* **> 2000**	**350** *600* *1000* *1400*		750* *1400*	
MON87701 soybean	**+**				**+**			
	**A**	**B**	**C**	**D**	**A**	**B**	**C**	**D**
	**550**	*450* *530*	*500* **1000** *> 2000*	**430** 1000* *1700* *> 2000*	*320* **450** *550* *860*	*550* *700* *1000* 1800*	*550* *660* *700* *1000*	*300* *470* *850*
KeFeng-6 rice	**+**				**+**			
	**A**	**B**	**C**	**D**	**A**	**B**	**C**	**D**
	**550**	*440* *520* *720* *970*	*250* *470* *950* *790*	**430** 980* **1650**	**700** *1000*	**500** **750** **1100**	*370* *500* *800* *1200*	950* **1500** **1800**
MON15985 cotton	**+**				**+**			
	**A**	**B**	**C**	**D**	**A**	**B**	**C**	**D**
	**550**	**500**	900*	**430** *800* *1000* *> 1500*	**350** **600** **1000** *1500*	*> 2000*	*500* 800* *1000* *1200*	**220**
281–24-236 × 3006–210-23 cotton	**+**				**+**			
	**A**	**B**	**C**	**D**	**A**	**B**	**C**	**D**
	*200* *450* *900* *1000*		*550*	420*	*300* **550**	*520*	*550* 750*	
MON89034 maize	**+**				**+**			
	**A**	**B**	**C**	**D**	**A**	**B**	**C**	**D**
	**600** *850* 1000*	*660*		**420** *500* *650*	*900*	300* *600*	*550*	*750*
Bt176 maize	**+**				**+**			
	**A**	**B**	**C**	**D**	**A**	**B**	**C**	**D**
	*550*	*250*	*600*	400* *550* *670* *1000*	*350* *500*	*300* *600*	*350* 450*	*500* **850** *1000* *1700*

For each result, the experiment was carried out in triplicate. The detection of the targets is symbolized by + (amplicons observed in each repetition) or (+) (amplicons not observed in each repetition). The approximate size of amplicons is indicated in base-pair for each DRT primers. The amplicons observed in each repetition (3/3) are indicated in bold while the amplicons obtained not in each repetition (1–2/3) are indicated in italic. The sequenced amplicons are indicated by an asterisk

All the characterized sequences started specifically to the targeted CryAb/c sequence region for all tested GM events. The sequences generated with the Cry-F DNA walking direction presented for all tested GM events the continuity of the Cry1Ab, Cry1Ac or Cry2Ab2 gene. The sequences observed for the Cry-R DNA walking direction presented for all tested GM events an unnatural association between the Cry1Ab, Cry1Ac or Cry2Ab2 gene and others transgenic elements (Fig. 1; Additional file 3). All these sequences, including either unnatural associations of transgenic elements or synthetic Cry genes that are highly different compared to the Cry genes coming from *B. thurengensis*, demonstrate the presence of GMO in the tested samples [14, 15].

### Sensitivity assessment of the bidirectional DNA walking method anchored on the CryAb/c genes

In order to assess their sensitivity, the developed bidirectional DNA walking methods anchored on the CryAb/c genes were applied on samples containing 20 to 2000 HGE of three GM events, namely Bt11 maize, MON810 maize and T304–40 cotton, possessing in their transgenic cassette the targeted CryAb/c sequences (Table 3). The tested GM events were selected as they presented no (Bt11) or few (MON810 and T304–40) nucleotide variations regarding the annealing sites of the target-specific primers (Fig. 1, Table 1, Additional file 1).

**Table 3:** Sensitivity analysis of the bidirectional DNA walking method, including the Cry-F and Cry-R walking direction, using the four different mixes of DRT primers (A-D)

	**Cry-F**											
	**2000 HGEs**				**200 HGEs**				**20 HGEs**			
Bt11 maize	**+**				**+**				**+**			
	**A**	**B**	**C**	**D**	**A**	**B**	**C**	**D**	**A**	**B**	**C**	**D**
	550	500	*650* *950* *1250*	*450* 1000 1400* 1800	*550*	*500*		*450* 1000 1400* 1800	*550*			1000* *1400* *1800*
MON810 maize	**+**				**+**				**+**			
	**A**	**B**	**C**	**D**	**A**	**B**	**C**	**D**	**A**	**B**	**C**	**D**
	550* *850* *1300*	*650*		400				400*			*1400*	400*
T304–40 cotton	**+**				**+**				**+**			
	**A**	**B**	**C**	**D**	**A**	**B**	**C**	**D**	**A**	**B**	**C**	**D**
	550 *800*	*350*	*450* *500* *600* 800* *1200*	400 *600*	550* *800* *1000*	*500* *1000*	*450* *500* *600* *800* *1200*	400 *500* *1000* *1700*	*250* 550*	*500*		400
	**Cry-R**											
Bt11 maize	**+**				**+**				**+**			
	**A**	**B**	**C**	**D**	**A**	**B**	**C**	**D**	**A**	**B**	**C**	**D**
	400* *800*	*> 2000*	*500* *1000* *1400*	*550* *1300* *1600*	*300* 400*	*> 2000*	*1200*	*300* *550* *1700*	*300* 400*		*500* *1000*	*550* *1000*
MON810 maize	**+**				**+**				**+**			
	**A**	**B**	**C**	**D**	**A**	**B**	**C**	**D**	**A**	**B**	**C**	**D**
	250*	*770*	*350* 500	*260*	250*	*600*	*500*	*350*	250*	*350*		*350*
T304–40 cotton	**+**				**+**				**(+)**			
	**A**	**B**	**C**	**D**	**A**	**B**	**C**	**D**	**A**	**B**	**C**	**D**
	*550*	*200*	*200* *450* *550*	*200* *450* 700*	*250* *550*	200* *1000*	*550*	*250* *450*	*250*	200*	*550*	*450*

For each tested sample (Bt11, MON810 and T304–40), 2000, 200, 20 HGEs of the target were tested. For each result, the experiment was carried out in triplicate. The detection of the targets is symbolized by + (amplicons observed in each repetition) or (+) (amplicons not observed in each repetition). The approximate size of amplicons is indicated in base-pair for each DRT primers. The amplicons observed in each repetition (3/3) are indicated in bold while the amplicons obtained not in each repetition (1–2/3) are indicated in italic. The sequenced amplicons are indicated by an asterisk

With the Cry-F DNA walking direction, several amplicons, with a size ranging from 250 bp to 1.8 Kbp, were generated for each tested GM event at each tested target concentration. The characterization of the sequenced amplicons allowed to identify the sequence of continuity of the Cry1Ab genes [GenBank: GU583854, AY326434, KJ716235] (Table 3, Additional file 3). Using the Cry-R DNA walking direction, several amplicons, with a size range going from 200 bp to more than 2 Kbp, were generated for the Bt11, MON810 and T304–40 events at a GM target concentration of 2000, 200 and 20 HGE and allowed to characterize the unnatural association between the Cry1Ab gene and an Intron [GenBank: EU363767, AY562548, AY326434, KJ716235, CP018157]. However, at the concentration of 20 HGE, the amplicons from the T304–40 event were not observed for each performed assays (Table 3, Additional file 3) although no nucleotide variation was observed in the targeted Cry1Ab region from the T304–40 event (Additional file 4).

### Applicability of the bidirectional DNA walking method anchored on the Cry1Ab/c genes

To illustrate the applicability of the proposed bidirectional DNA walking method anchored on the CryAb/c genes, a processed food matrix (GeM MP17), originated from a GeMMA Scheme Proficiency Test, was used to mimic a real-life sample encountered in GMO routine analysis. This processed food matrix was a GMO mixture composed of two GM maize events (MON863 and MON810) present in low amounts (respectively 4.30 and 6.95%). One of these GM maize events, MON810, possesses in its transgenic cassette the Cry1Ab gene.

Following to a positive signal for the qPCR Cry1Ab/c screening marker (i.e., Cq: 29.6; Tm: 78.50 °C) [13], the bidirectional DNA walking method anchored on the CryAb/c genes was applied on this processed food matrix. For each DNA walking direction, only one amplicon, chose for its large size as well as for its ease to be selected on an electrophoresis gel, was sequenced. By comparison to publicly available databases, the sequence generated by the Cry-F DNA walking direction was corresponding to the Cry1Ab gene [GenBank: AY326434], which is found in several insect-resistant GM events (Additional file 5) [14]. The characterization of the sequence obtained with the Cry-R DNA walking direction presented an unnatural association of transgenic elements composed of a part of the Cry1Ab gene followed by a part of the hsp70 intron [GenBank: AY326434] (Additional file 5). In addition, this unnatural association was corresponding, as expected, to the GM MON810 event, according to the patent US 9562267 B2.

## Discussion

With the aim to propose an integrated approach to the GMO routine analysis, the couple of primers targeting the CryAb/c elements in the qPCR screening step was also used as target-specific primers in the DNA walking step developed in this study [13]. An additional primer was designed on the CryAb/c genes to fulfil the criteria required by the DNA walking method (Fig. 1; Table 1). The developed and validated bidirectional DNA walking method anchored on the CryAb/c genes was successfully applied on several GM events (Bt11, MON810, MON89034, Bt176, T304–40, MON531, 281–24-236 × 3006–210-23, MON15985, MON87701 and KeFenkg-6) possessing the targeted sequences. All sequenced amplicons generated from these GM events were corresponding either to a part of their transgenic cassette (unnatural associations of elements), using the Cry-R DNA walking direction, or to synthetic Cry gene sequences, using the Cry-F DNA walking direction. These results allowed confirming the presence of GMO in the tested samples. Moreover, the impact of the used DNA walking directions regarding the type of generated sequences was expected due to the position of the target-specific primers nearby one extremity of the CryAb/c gene sequences.

Based on its sensitivity assessment, the proposed bidirectional DNA walking method anchored on the CryAb/c genes has showed to be able to deal with low amounts of targets (as low as 20 HGE). However, at the concentration of 20 HGE, a difference in the sensitivity performance has been observed using the Cry-R DNA walking direction since amplicons were observed for the MON810 and Bt11 events while no amplicon was observed for the T304–40 event for each performed assays (Table 3, Additional file 3). Given that no nucleotide variation was observed in the targeted Cry1Ab region from the T304–40 event (between the sequenced sequence from the PCR verification assay and the reference sequence used for the design of the target-specific primers) (Additional file 4), this performance drop could only be related to a weaker affinity of the DRT primers used and could thus be solve by the used of additional DRT primer mixes. The sensitivity performance may thus vary from one GM event to another one in function to the targeted amplified sequences.

The applicability of the proposed bidirectional DNA walking method anchored on the CryAb/c genes was tested using a processed food matrix mimicking a real-life sample encountered in GMO routine analysis. The characterized sequences corresponded either to the synthetic Cry1Ab gene or to a part of the synthetic Cry1Ab gene followed by a part of the hsp70 intron (Additional file 5). Given that this synthetic Cry gene has been widely used to produce insect-resistant GM events as well as its sequence is highly different compared to the Cry gene coming from *B. thurengensis*, the presence of an insect-resistant GMO in the tested sample was very likely [14]. This hypothesis was supported by the characterization of an unnatural association of transgenic elements (Additional file 5), allowing to prove the presence of GMO in the tested sample. In addition, the added value to compare the generated data to available public databases containing patents was highlighted since, this way, the identity the GM event (MON810) was revealed.

## Conclusion

A new DNA walking starting point on the CryAb/c genes, with two walking directions, has been here developed and validated to strengthen the recently published integrated DNA walking strategy [10–12, 16]. According to a sensitivity assessment, this bidirectional DNA walking method has showed to be able to deal with low amounts of targets (as low as 20 HGE) and to be successfully applied on a processed sample mimicking food/feed matrices that are commonly encountered in GMO routine analysis. On this basis, this new bidirectional DNA walking method anchoring on the CryAb/c genes can be integrated to the set of key targets (p35S, tNOS and t35S pCAMBIA) described in the recently proposed strategy used to demonstrate the presence of unauthorized GMO in the food and feed chain [3, 10–12, 16].

As previously described, this strategy includes first the detection of the potential presence of GMO by qPCR screening in targeting key elements (p35S, tNOS, t35S pCAMBIA and CryAb/c), which is performed in GMO routine analysis by enforcement laboratories. In targeting these four elements, approximately 70% of EU authorized GMO and 95% of EU unauthorized GMO are covered [9–12, 16]. In case of positive signals for at least one of these key elements, this step is followed by the DNA walking methods anchored on these detected elements and the sequencing of the produced amplicons. The analysis of the generated sequences may reveal transgene flanking regions (unique for each GM event) and unnatural associations of transgenic elements, allowing to identify the presence of GMO. The presence of EU unauthorized GMO will be highlighted by transgene flanking regions, unnatural associations of elements and synthetic sequences (i.e., synthetic Cry genes) that are not found in EU authorized GMO [3, 10–12, 16].

In the context of GMO routine analysis, the sequencing of all generated amplicons from each DNA walking method is advised in order to guarantee the entire representativeness of several GMO potentially present in the tested food/feed matrix. In this regard, the entire final PCR products from each DNA walking method can be sequenced using long-range Next-Generation-Sequencing (NGS) platforms. This way, we avoid the individual selection of all generated amplicons from the electrophoresis gel and their individual subsequent sequencing using the Sanger technology, which can be a laborious work. Given its simple procedure (PCR-based method using the same target-specific primers for the qPCR screening and the DNA walking steps) and its short time-frame to get results, this strategy could therefore easily be implemented in GMO routine analysis by the enforcement laboratories [3, 10–12, 16].

## Methods

### Plant material

The CRMs of the GM maize Bt11–5% (ERM-BF412f), MON810–9.9% (ERM-BF413gk), MON89034–100% (AOCS 0906-E), and Bt176–5% (ERM-BF411f), the GM cotton T304–40-10% (ERM-BF429c), MON531–100% (AOCS 0804-C), 281–24-236 × 3006–210–23-100% (ERM-BF422b), and MON15985–100% (AOCS 0804-D), and the GM soybean MON87701–100% (AOCS 0809-A), in the form of seed powder or genomic DNA (gDNA), were obtained from the American Oil Chemists’ Society and the Institute for Reference Materials and Measurements (AOCS, Urbana, USA; IRMM, Geel, Belgium). The CRM of the GM rice KeFenkg-6-5%, in the form of gDNA was obtained from the Europe Union Reference Laboratory for GM Food and Feed (EU-RL GMFF, JRC, Ispra, Italy). The processed food matrix (GeM MP17), coming from a GeMMA Scheme Proficiency Test, was prepared by blending wild-type (WT) soybean, maize and wheat flour with GM maize MON810–100% flour and GM maize MON863–100% flour. The blend was mixed with water to create dough that has been baked to be then milled into powder. The final amount (*w*/w) of the GM maize MON810 and MON863 was respectively 6.95 and 4.30% (www.fapas.com).

### DNA extraction, concentration and purity

Using a CTAB-based procedure (ISO 21571), DNA was extracted from CRMs and food matrix (GeM MP17) as previously described (Broeders et al., 2012). Using the Nanodrop® 2000 (ThermoFisher, DE, USA) device, DNA concentration was measured by spectrophotometry and the DNA purity was evaluated using the A260/A280 and A260/A230 ratios.

### qPCR assay

The qPCR assay applied on 25 ng of DNA from the GeM MP17 sample was performed using the Cry1Ab/c qPCR screening marker [13]. The qPCR program consisted of initial DNA polymerase activation for 10 min at 95 °C followed by 40 amplification cycles of 95 °C for 15 s (denaturing step) and 60 °C for 1 min (annealing-extension step). Melting curve analyses were performed by gradually increasing the temperature from 60 °C to 95 °C in 20 min (±0.6°/20 s). The run was performed on a CFX96 Touch Real-Time PCR Detection System (BioRad, Hemel Hempstead, UK). For each assay, a “no template control” was included.

### DNA walking methods

#### Development of target-specific primers in silico

The DNA walking approach has been developed to anchor on the region targeted by the Cry1Ab/c qPCR screening marker which is found in the synthetic Cry1Ab, Cry1Ac and Cry2Ab2 genes (namely here CryAb/c genes) [13]. From the targeted CryAb/c transgenic elements, two walking directions, called forward (F) and reverse (R), have been performed. For each walking direction, three target-specific primers are required to carry out first the DNA walking (a) and then the first (b) and the second (c) semi-nested PCR rounds. To provide an integrated approach, the design of two target-specific primers, b and c for the forward walking direction and a and b for the reverse walking direction, is based on the sequences from the Cry1Ab/c SYBR®Green real-time PCR screening marker published by Barbau-Piednoir et al., 2012 (Fig. 1; Table 1) [13]. To design the third primer, corresponding to the target-specific primer a for the forward walking direction and the target-specific primer c for the reverse walking direction, the targeted CryAb/c sequences from the GM events positive for the Cry1Ab/c SYBR®Green real-time PCR screening markers (Bt11, MON87701, MON87751, MON531, Bt176, T304–40, Bt63, MON810, KeFeng-6, MON89034, MON15985, 281–24-236 × 3006–210-23), originating from an in-house database, public databases and patents, have been compared using the program “Clustal Omega”. On this basis, the third primer was then designed, with the help of the program “Primer3”, in taking into account the nucleotide variations of the targeted region between the GM events positive for the Cry1Ab/c qPCR screening marker (Fig. 1; Table 1; Additional file 1) [13]. The specificity of this new primer was successfully assessed in silico, against all EU-authorized GMOs, LLPs (Low Level Presence) and corresponding WT, using the software wEMBOSS.

#### DNA walking and double semi-nested PCR reactions

The DNA walking strategy previously described by Fraiture et al., 2014 was adapted here to anchor on the region targeted by the Cry1Ab/c qPCR screening marker [10, 13]. In the same way, a first reverse target-specific primer (a) and one kind of the degenerated random tagging (DRT) primer mix (A-D) were first applied (Additional file 6). This step was then followed by two semi-nested PCR rounds using target-specific primers (b and c), that are each time nested to the previous reverse target-specific primer, combined to universal tagging primers (UAP-N1 and UAP-N2) (Additional file 6). The bidirectional DNA walking method anchored on the CryAb/c genes was applied on 2000, 200 and 20 HGE of the Bt11, MON810 and T304–40 events as well as on 2000 HGE of the MON531, MON87701, MON15985, KeFeng-6, MON89034, Bt176 and 281–24-236 × 3006–210-23 events. This bidirectional DNA walking method was also applied on 100 ng of DNA from the processed food matrix (GeM MP17). Moreover, a NTC was included for each assay. PCR mixes and conditions were carried out according to the manufacturer’s instructions (APAgene™ GOLD Genome Walking Kit from BIO S&T, Montréal, Canada). The final PCR products were analysed by electrophoresis on a 1% agarose gel (INVITROGEN, CA, USA) (100 V, 400 mA, 60 min) in view to further analysis allowing to identify the generated sequences. The final PCR products were also analysed by electrophoresis using the Tapestation 4200 device with the associated High Sensitivity D5000 Screen Tape and reagents (Agilent, Belgium) in order to visualize the profiles of generated amplicons (Additional file 2).

#### Sequencing

For each DNA walking method, only the longest and easily selectable amplicon observed for each DRT primer mix was excised from agarose gel and purified using the QIAEX II Gel Extraction Kit (QIAGEN, Hilden, Germany), according to the manufacturers’ instructions, to be sequenced on a Sanger sequencing platform using the corresponding target-specific c primer. All sequencing reactions were performed on a Genetic Sequencer 3130XL using the Big Dye Terminator Kit v3.1 (Applied Biosystems, CA, USA). The sequences were analysed using publicly available databases, including the “Nucleotide BLAST NCBI” software, the “JRC GMO-Amplicon” tool (http://gmo-crl.jrc.ec.europa.eu/jrcgmoamplicons/db_scans/blast) and/or a patent database (https://www.lens.org/lens/).

### Verification of the targeted cry region from the T304–40 event

To verify the correctness of the sequence from T304–40 used to design the target-specific primers, a PCR assay was carried out to amplify the sequence region where the target-specific primers were designed. To this end, a couple of primers was designed using the program “Primer3” (Table 1). A standard 25 μl reaction volume was applied containing 12.5 μl of DreamTaq Green PCR Master Mix (ThermoFisher Scientific, Merelbeke, Belgium), 250 nM of each primer and 5 μl of T304–40-10% DNA (20 ng/μl). The PCR program consisted of a single cycle of 1 min at 95 °C (initial denaturation) followed by 35 amplification cycles of 30 s at 95 °C (denaturation), 30 s at 55 °C and 1 min at 72 °C (extension) and finishing by a single cycle of 5 min at 72 °C (final extension). The run was performed on a Swift MaxPro Thermal Cycler (Esco, Analis, Rhisnes, Belgium). The generated amplicon was analysed by electrophoresis on a 1% agarose gel (100 V, 400 mA, 60 min; INVITROGEN, CA, USA) and purified using the QIAEX II Gel Extraction Kit (QIAGEN, Hilden, Germany), according to the manufacturers’ instructions, to be sequenced. All sequencing reactions were performed on a Genetic Sequencer 3130XL using the Big Dye Terminator Kit v3.1 (Applied Biosystems, CA, USA). The obtained sequence was compared to the sequence from T304–40 used to design the target-specific primers using the software “Clustal Omega” (Additional file 4).

## Additional files

Additional file 1: The nucleotide variations, indicated by the red crosses, between the designed target-specific primers (Cry-F a, b and c) and the targeted sequences GM events possessing the CryAb/c elements (Bt11, MON87701, MON87751, MON531, Bt176, T304–40, Bt63, MON810, KeFeng-6, MON89034, MON15985 and 281–24-236 × 3006–210-23 events). (DOCX 15 kb)

Additional file 2: Visualization of the PCR products from the bidirectional DNA walking method anchored on CryAb/c applied on 2000 HGE of GM events (Bt11, MON87701, MON87751, MON531, Bt176, T304–40, Bt63, MON810, KeFeng-6, MON89034, MON15985 and 281–24-236 × 3006–210-23 events). (DOCX 1205 kb)

Additional file 3: Sequences of the sequenced amplicons (see Tables 2 and 3). (DOCX 20 kb)

Additional file 4: Alignement of the targeted Cry1Ab sequences from the T304–40 event sequenced from the PCR verification assay (1 and 2) with the reference sequence from the T304–40 event (reference) used for the design of the target-specific primers (surrounded by orange rectangles). (DOCX 506 kb)

Additional file 5: Sequences of the sequenced amplicons from the processed food matrix (GeM MP17) coming from a GeMMA Scheme Proficiency Test. (DOCX 14 kb)

Additional file 6: Sequences of oligonucleotides provided by the APAgeneTM GOLD Genome Walking Kit from BIO S&T. (DOCX 13 kb)
