# Supplementary material for: Development and validation of an integrated DNA walking strategy to detect GMO expressing cry genes
Source: BMC Biotechnol. 2018 Jun 27;18:40. doi: 10.1186/s12896-018-0446-x (PMC6020286; doi:10.1186/s12896-018-0446-x)
Supplement: Supplementary file 1 — The nucleotide variations, indicated by the red crosses, between the designed target-specific primers (Cry-F a, b and c) and the targeted sequences GM events possessing the CryAb/c elements (Bt11, MON87701, MON87751, MON531, Bt176, T304–40, Bt63, MON810, KeFeng-6, MON89034, MON15985 and 281–24-236 × 3006–210-23 events). (DOCX 15 kb) [file 12896_2018_446_MOESM1_ESM.docx]

**Additional file 1: Nucleotide variations, indicated by the red crosses, between the designed target-specific primers (Cry-F a, b and c) and the targeted sequences GM events possessing the CryAb/c elements (Bt11, MON87701, MON87751, MON531, Bt176, T304-40, Bt63, MON810, KeFeng-6, MON89034, MON15985 and 281-24-236 x 3006-210-23 events).**

|  | **Cry-F a** | **Cry-F b** | **Cry-F c** |
| --- | --- | --- | --- |
| **Bt11 maize** | TGCATTCCATACAACTGCTTGAG | ACCGGTTACACTCCCATCGA | GAGTTCGTGCCAGGTGCTG |
| **MON87701 soybean** | TGCATTCCATACAACTGCTTGAG | ACCGGTTACACTCCCATCGA | GAGTTCGTGCCAGGTGCTG |
| **MON87751 soybean** | TGCATTCCATACAACTGCTTGAG | ACCGGTTACACTCCCATCGA | GAGTTCGTGCCAGGTGCTG |
| **MON531 cotton** | TGCATTCCATACAACTGCTTGAG | ACCGGTTACACTCCCATCGA | GAGTTCGTGCCAGGTGCTG |
| **MON15985 cotton** | TGCATTCCATACAACTGCTTGAG | ACCGGTTACACTCCCATCGA | GAGTTCGTGCCAGGTGCTG |
| **281-24-236 x 3006-210-23 cotton** | TGCATTCCXTACAACTGCXTGAG | ACXGGTTACACXCCXATCGA | GAGTTCGTGCCXGGTGCTG |
| **Bt63 rice** | TGCAXXCCATACAACTGCTTGAG | ACCGGTTACACTCCCATCGA | GAGTTCGTGCCAGGTGCTG |
| **T304-40 cotton** | TGCATXCCXTACAACTGCXTGAG | ACCGGTTACACXCCCATCGA | GAGTTCGTGCCXGGTGCTG |
| **Bt176 maize** | TGCATXCCXTACAACTGCXTGAG | ACCGGXTACACXCCCATCGA | GAGTTCGTGCCXGGXGCXG |
| **MON89034 maize** | TGCATXCCXTACAACTGCXTXAG | ACCGGTTACACXCCCATCGA | GAGTTCGTGCCAGGXGCTG |
| **MON810 maize** | TGCATXCCXTACAACTGCXTXAG | ACCGGTTACACXCCCATCGA | GAGTTCGTGCCAGGXGCTG |
| **KeFeng-6 rice** | Not available | Not available | Not available |
