# Supplementary material for: Development and validation of an integrated DNA walking strategy to detect GMO expressing cry genes
Source: BMC Biotechnol. 2018 Jun 27;18:40. doi: 10.1186/s12896-018-0446-x (PMC6020286; doi:10.1186/s12896-018-0446-x)
Supplement: Supplementary file 2 — Visualization of the PCR products from the bidirectional DNA walking method anchored on CryAb/c applied on 2000 HGE of GM events (Bt11, MON87701, MON87751, MON531, Bt176, T304–40, Bt63, MON810, KeFeng-6, MON89034, MON15985 and 281–24-236 × 3006–210-23 events). (DOCX 1205 kb) [file 12896_2018_446_MOESM2_ESM.docx]

**Additional file 2: Visualization of the PCR products from the bidirectional DNA walking method anchored on CryAb/c applied on 2000 HGE of GM events (Bt11, MON87701, MON87751, MON531, Bt176, T304-40, Bt63, MON810, KeFeng-6, MON89034, MON15985 and 281-24-236 x 3006-210-23 events). The ladder indicates a size range going from 15 to 5000 bp. For each GM event, the different DRT primer mixes are indicated from A to D. These results were obtained by the analysis of the final PCR products using the Tapestation 4200 device.**

|  | **Cry-F** | **CryR** |
| --- | --- | --- |
| **Bt11 maize** | **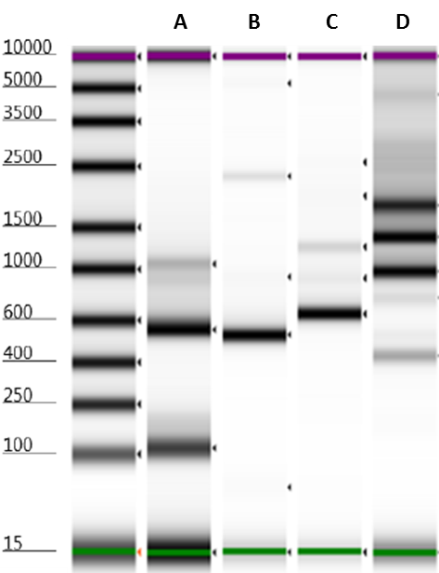** | **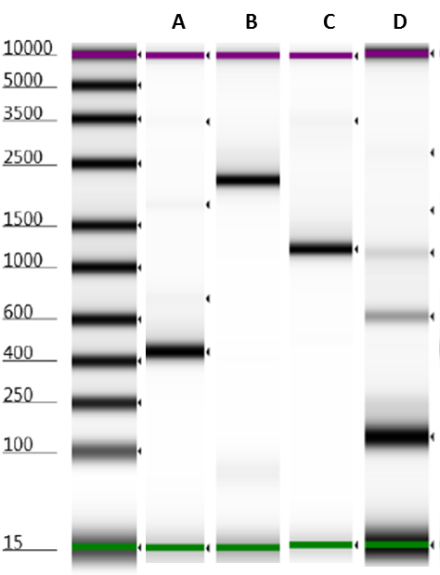** |
| **MON810 maize** | **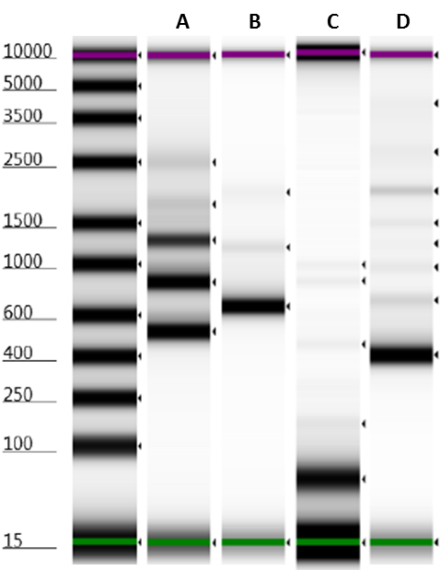** | **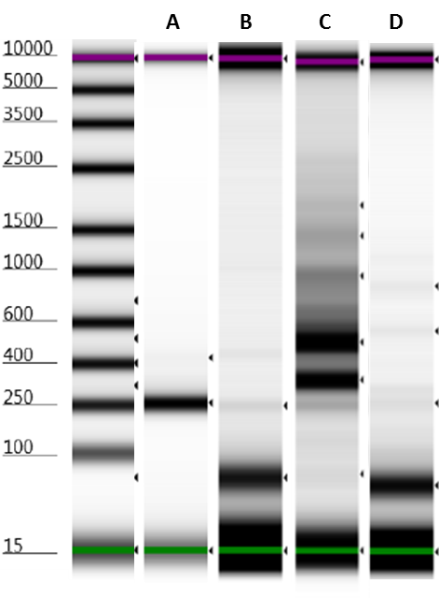** |
| **T304-40 cotton** | **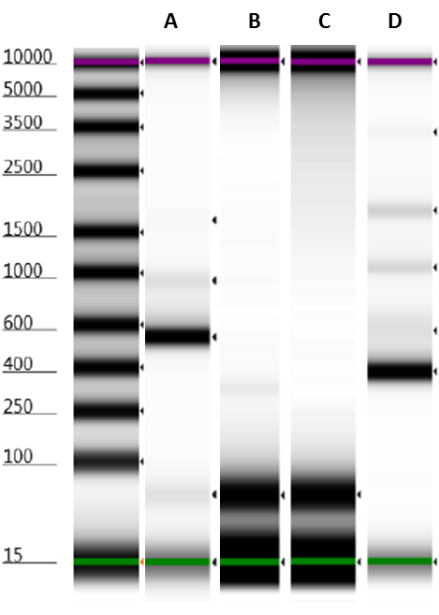** | **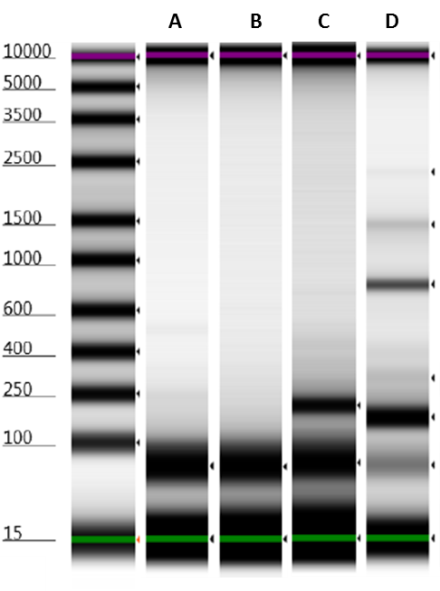** |
| **MON531 cotton** | **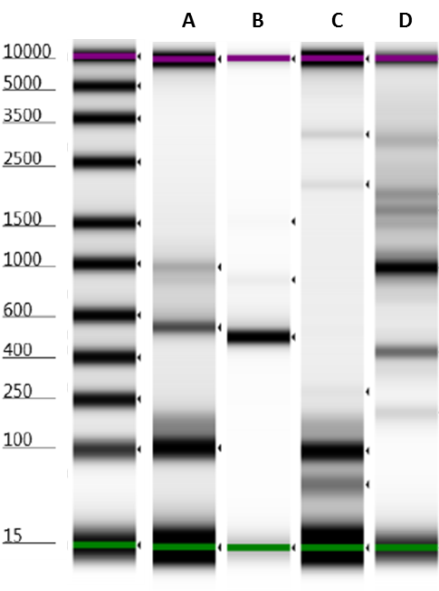** | **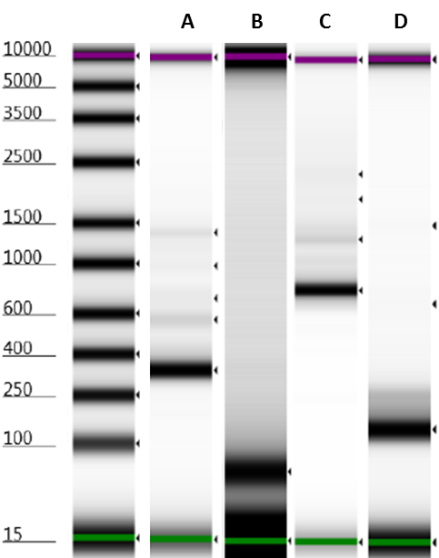** |
| **MON87701 soybean** | **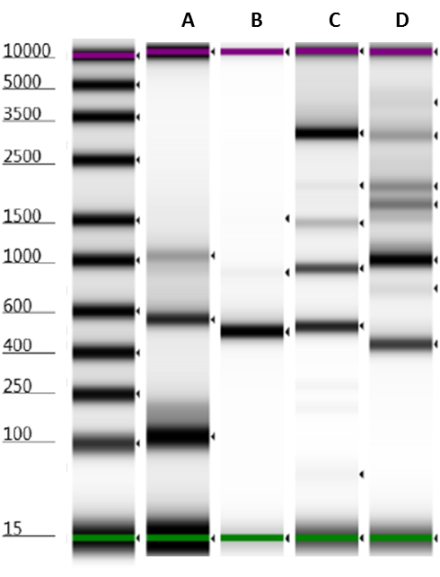** | **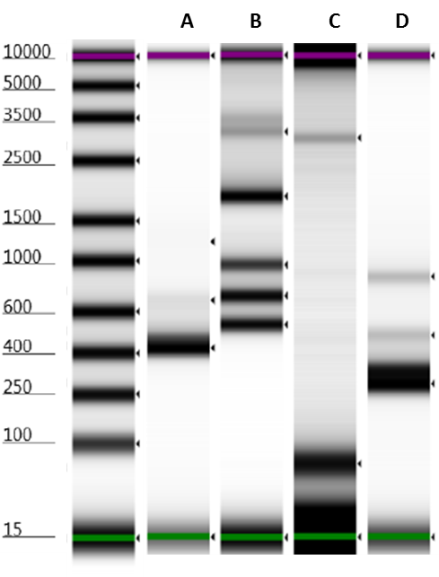** |
| **KeFeng-6 rice** | **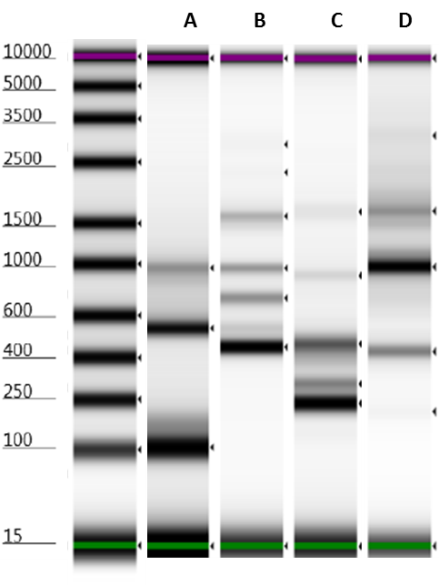** | **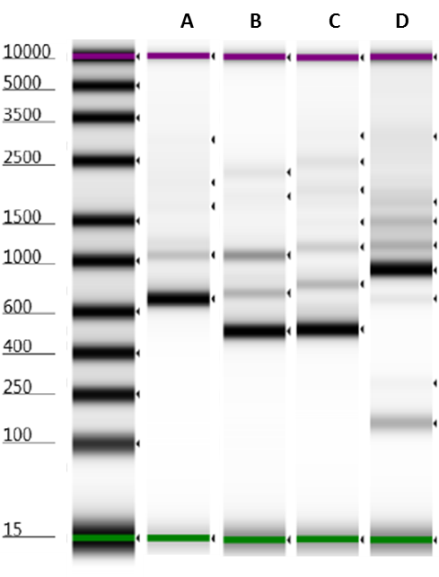** |
| **MON15985 cotton** | **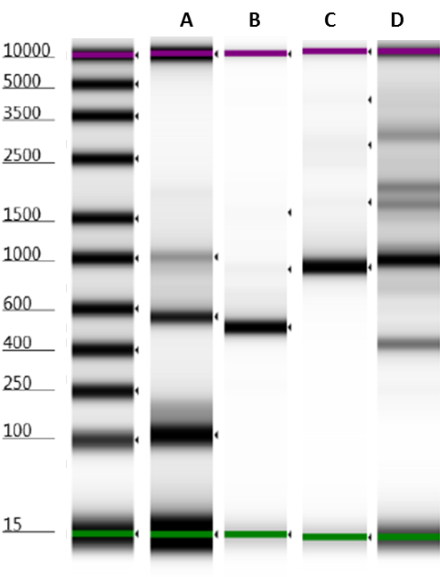** | **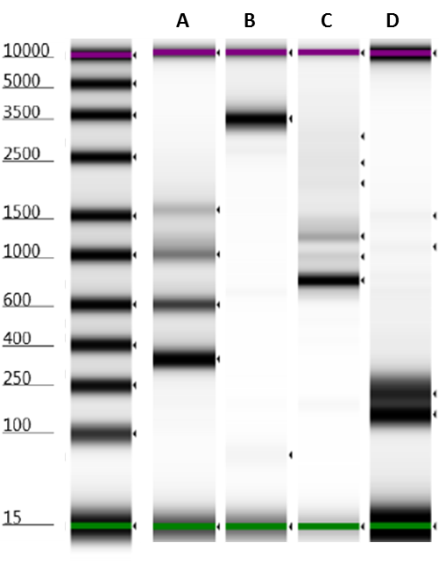** |
| **281-24-236 x 3006-210-23 cotton** | **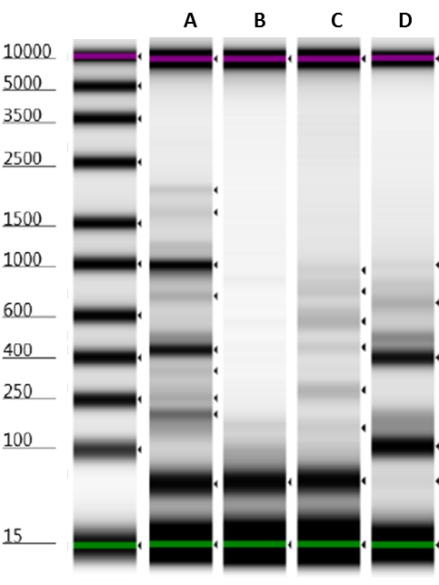** | **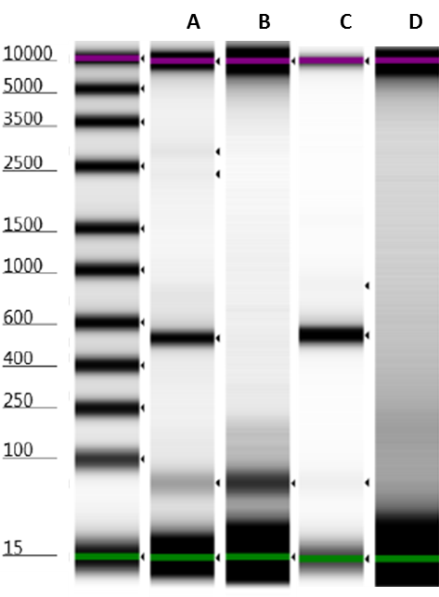** |
| **MON89034 maize** | **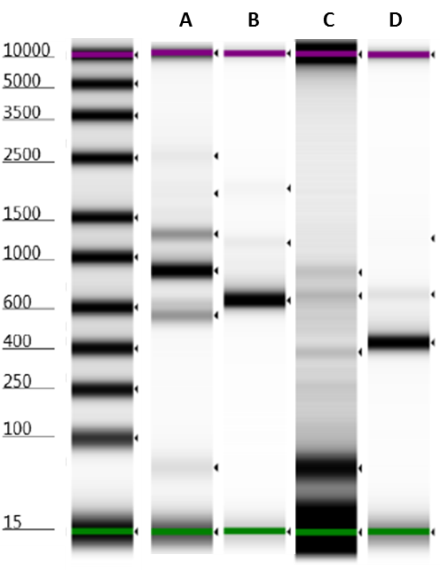** | **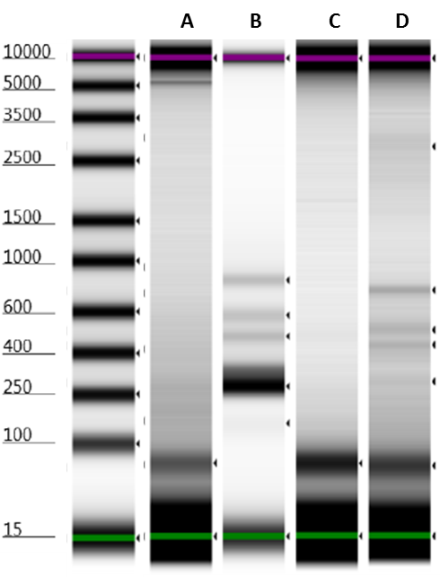** |
| **t176 maize** | **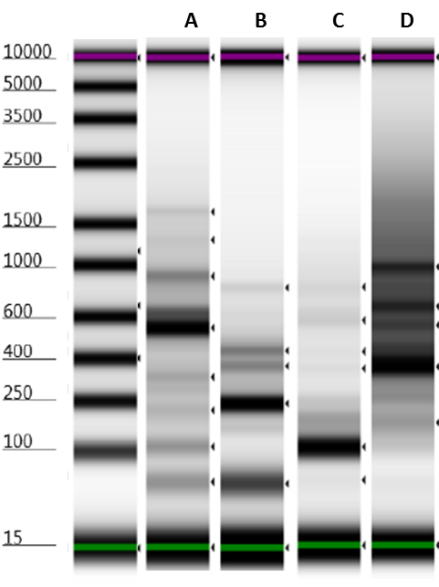** | **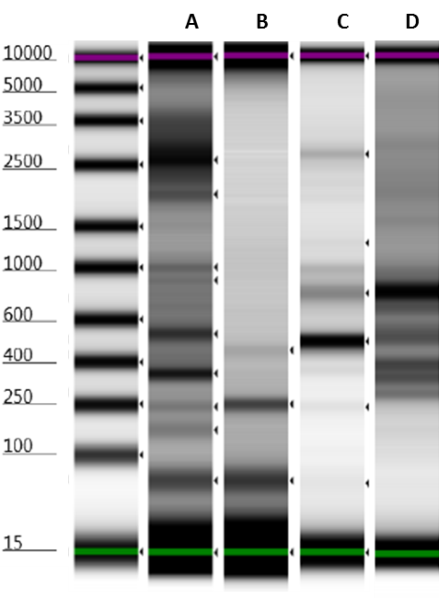** |
