# Supplementary material for: Development and validation of an integrated DNA walking strategy to detect GMO expressing cry genes
Source: BMC Biotechnol. 2018 Jun 27;18:40. doi: 10.1186/s12896-018-0446-x (PMC6020286; doi:10.1186/s12896-018-0446-x)
Supplement: Supplementary file 3 — Sequences of the sequenced amplicons (see Tables 2 and 3). (DOCX 20 kb) [file 12896_2018_446_MOESM3_ESM.docx]

**Additional file 3: Sequences of the sequenced amplicons (see Table 2 and 3).**

**CryAb/c genes (italic); Intron (underlined); Transit peptide (wave underline); Promoter of the *Arabidopsis thaliana* RbcS4 gene encoding ribulose 1,5-bisphosphate carboxylase small subunit 1A (pRbcs4) (bold); Promoter of the polyubiquitin gene (pUBI) (double underlined); cow pea trypsin inhibitor gene (CpTi) (lowercase); Terminator of the nopaline synthase gene from Agrobacterium tumefaciens (tNOS) (lowercase bold); Promoter 35S of the Cauliflower Mosaic Virus (CaMV) (p35S) (dotted underlined); Part of Zea mays calcium-dependent protein kinase gene (lowercase wave underline).**

| >Bt11_Cry-F: Cry1Ab [GenBank: EU816953]  *TCATCTGGGGTATCTTTGGTCCATCTCAATGGGATGCATTCCTAGTGCAAATTGAGCAGTTGATCAACCAGAGGATCGAAGAGTTCGCCAGGAACCAGGCCATCTCTAGGTTGGAAGGATTGAGCAATCTCTACCAAATCTATGCAGAGAGCTTCAGAGAGTGGGAAGCCGATCCTACTAACCCAGCTCTCCGCGAGGAAATGCGTATTCAATTCAACGACATGAACAGCGCCTTGACCACAGCTATCCCATTGTTCGCAGTCCAGAACTACCAAGTTCCTCTCTTGTCCGTGTACGTTCAAGCAGCTAATCTTCACCTCAGCGTGCTTCGAGACGTTAGCGTGTTTGGGCAAAGGTGGGGATTCGATGCTGCAACCATCAATAGCCGTTACAACGACCTTACTAGGCTGATTGGAAACTACACCGACCACGCTGTTCGTTGGTACAACACTGGCTTGGAGCGTGTCTGGGGTCCTGATTCTAGAGATTGGATTAGATACAACCAGTTCAGGAGAGAATTGACCCTCACAGTTTTGGACATTGTGTCTCTCTTCCCGAACTATGACTCCAGAACCTACCCTATCCGTACAGTGTCCCAACTTACCAGAGAAATCTATACTAACCCAGTTCTTGAGAACTTCGACGGTAGCTTCCGTGGTTCTGCCCAAGGTATCGAAAGGCTCCATCAGGAGCCCACACTTGATGGACATCTTGAACAGCATAACTATCTACACCGATGCTCACAGAGGAGAGTATTACTG*  >Bt11_Cry-R: Cry1Ab followed by an intron sequence [GenBank: EU363767, AY562548]  *GTCCATG*GTCGACTCTAGAGGATCCGCGGCTTGTTGTGGTCTTTTGGGTTCACAAATTCACGTGCAACCGAACTTCCTAGCTGAAAAATGGGACAGATGGATGAGCTACTGACTTGTCGAAACAATGTAATAAAATGACTAATCAGTTATTAGTCCAAGGCCAGTGCAACCAAACTTCCTAGCTGAAAAAATGGGACAGATGGATGAGCTACTGACTTGTCGAAACAACGTAATAAAATGACTACTCAGTTATTAGACCAAGGCCAGTGCAACCAAACTTCCAAGCTGAAAAAATGGGACAAATGGATGAACTACTGACTTGTCAAAACAAAGTAATAAAATGACTACTCAGTGATTATACCAAGGCATGCCTTCAATCGAAGGGATACATCATACATATATACATGTGTGTGTACTGTACCTTCTTCGAATCTGCTGGGGTTCAGGGCG  >MON810_Cry-F: Cry1Ab [GenBank: AY326434]  *TCATCTGGGGCATCTTTGGCCCCTCCCAGTGGGACGCCTTCCTGGTGCAAATCGAGCAGCTCATCAACCAGAGGATCGAGGAGTTCGCCAGGAACCAGGCCATCAGCCGCCTGGAGGGCCTCAGCAACCTCTACCAAATCTACGCTGAGAGCTTCCGCGAGTGGGAGGCCGACCCCACTAACCCAGCTCTCCGCGAGGAGATGCGCATCCAGTTCAACGACATGAACAGCGCCCTGACCACCGCCATCCCACTCTTCGCCGTCCAGAACTACCAAGTCCCGCTCCTGTCCGTGTACGTCCAGGCCGCCAACCTGCACCTCAGCGTGCTGAGGGACGTCAGCGTGTTTGGCCAGAGGTGGGGCTTCGACGCCGCCACCATCAACAGCCGCTACAACGACCTCACCAGGCTGATCGGCAACTAC*  >MON810_Cry-R: Cry1Ab followed by an intron sequence [GenBank: AY326434]  *GTTGTTGTCCAT*GGCCGCTTGGTATCTGCATTACAATGAAATGAGCAAAGACTATGTGAGTAACACTGGTCAACACTAGGGAGAAGGCATCGAGCAAGATACGTATGTAAAGAGAAGCAATATAGTGTCAGTTGGTAGATACTAGATACCATCAGGAGGTAAGGAGAGCAACAAAAAGG  >T304-40_Cry-F: Cry1Ab [GenBank: AY326434]  *TCATCTGGGGCATCTTCGGTCCAGCCAGTGGGATGCCTTCCTGGTGCAGATCGAACAGTTAATTAACCAAAGAATAGAAGAATTCGCTAGGAACCAAGCCATCTCTAGACTGGAGGGCCTGAGCAACCTGTACCAGATCTACGCCGAGAGCTTCCGCGAGTGGGAGGCTGACCCCACCAACCCAGCCCTGCGCGAGGAGATGCGCATCCAGTTCAACGACATGAACTCTGCCCTGACCACCGCCATCCCACTCTTCGCTGTCCAGAACTACCAGGTCCCTCTCCTGTCTGTCTATGTGCAAGCTGCCAACCTCCATCTCAGCGTCCTTCGCGACGTGAGCGTCTTTGGGCAGAGGTGGGGGTTCGACGCTGCCACCATCAACAGCCGCTACAACGACCTGACGCGTCTGATCGGCAACTACACCGACCACGCAGTGAGATGGTACAACACTGGGCTTGAGAGGGTCTGGGGTCCCGACAGCCGCGACTGGATCAGGTACAACCAGTTCAGGCGTGAACTCACTCTCACCGTCTTGGATATCGTCAGTCTCTTCCCCAACTACGACAGCAGGACCTACCCTATCCGGACTGTGAGCCAGCTGACCCGCGAGATCTACACCAACCCCGTGCTGGAGAACTTCGACGGCAGCTTCAGGGGCTCTGCCCAGGGCATCGAGGGCAGCATCCGCAGCCCCCACCTGATGGACATCCTGA*  >T304-40_Cry-R: Cry1Ab followed by an intron sequence [GenBank: KJ716235, CP018157]  *CCATGGTTTTGG*CGCGACGGCTTGATGGATCTCTTGCTGGACACCGGGATGCTAGGATGCTCGAGTTAAAACCAGAACAGACGATGCGAAGAAGAATACG  >MON531_Cry-F: Cry1Ac [GenBank: KP195020]  *ATCATCTGGGTATCTTTGGTCCATCTCAATGGGATGCATTCCTGGTGCAATTGAGCAGTTGATCAACCAAAGGATCAAAAAGTTCCCCAGGAACCAGGCCTTCTCTAGGTTGAAAGGATTGACCATTCTCTACCAAATCTATGCAAAAAGCTTCAAAAAGGGGAAAGCCAATCCTACTAACCCACTTCTCCGCAAGGAAAGGCGAATTCATTTCAACAACTTGAACAGCGCCTTGACCACAGTTATCCCATTGTTCGCAGCCCGAAACTACCAGTTTCCTCTCTTGTCCGGGTACGTTCAAGCGGCTAATCTTCCCCTCGGCGGGCTTCAAAACTTTACCGGGTTGGGCCAAAGGGGGGAATTCATGGTTGCACCATTCATAACCCGTAACACCGACTTAACTGGGTTGATGGGAACTAC*  >MON531_Cry-R: Cry1Ac followed by p35S [GenBank: GU583853, KJ608145]  *TGTCATGGAGATCTGCTAGAGTCAGCTTGTCA*GCGTGTCCTCTCCAAATGAAATGAACTTCCTTATATAGAGGAAGGGTCTTGCGAAGGATAGTGGGATTGTGCGTCATCCCTTACGTCAGTGGAGATATCACATCAATCCACTTGCTTTGAAGACGTGGTTGGAACGTCTTCTTTTTCCACGATGCTCCTCGTGGGTGGGGGTCCATCTTTGGGACCACTGTCGGCAGAGGCATCTTCAACGATGGCCTTTCCTTTATCGCAATGATGGCATTTGTAGGAGCCACCTTCCTTTTCCACTATCTTCACAATAAAGTGACAGATAGCTGGGCAATGGAATCCGAGGAGGTTTCCGGATATTACCCTTTGTTGAAAAGTCTCAATCGGACCATCACATCAATCCACTTGCTTTGAAGACGTGGTTGGAACGTCTTCTTTTTCCACGATGCTCCTCGTGGGTGGGGGTCCATCTTTGGGACCACTGTCGGCAGAGGCATCTTCAACGATGGCCTTTCCTTTATCGCAATGATGGCATTTGTAGGAGCCACCTTCCTTTTCCACTATCTTCACAATAAAGTGACAGATAGCTGGGCAATGGAATCCGAGGAGGTTTCCGGATATTACCCTTTGTTGAAAAGTCTCAATCGGACCTGCAGCCAAGCTTGTCTGCAGTCGACGGATCCCCGGGAGCACAGATGACGCC  >MON87701_Cry-F: Cry1Ac [GenBank: KP195020]  *TCATCTGGGGTATCTTTGGTCCATCTCAATGGGATGCATTCCTGGTGCAAATTGAGCAGTTGATCAACCAGAGGATCGAAGAGTTCGCCAGGAACCAGGCCATCTCTAGGTTGGAAGGATTGAGCAATCTCTACCAAATCTATGCAGAGAGCTTCAGAGAGTGGGAAGCCGATCCTACTAACCCAGCTCTCCGCGAGGAAATGCGTATTCAATTCAACGACATGAACAGCGCCTTGACCACAGCTATCCCATTGTTCGCAGTCCAGAACTACCAAGTTCCTCTCTTGTCCGTGTACGTTCAAGCAGCTAATCTTCACCTCAGCGTGCTTCGAGACGTTAGCGTGTTTGGGCAAAGGTGGGGATTCGATGCTGCAACCATCAATAGCCGTTACAACGACCTTACTAGGCTGATTGGAAACTACACCGACCACGCTGTTCGTTGGTACAACACTGGCTTGGAGCGTGTCTGGGGTCCTGATTCTAGAGATTGGATTAGATACAACCAGTTCAGGAGAGAATTGACCCTCACAGTTTTGGACATTGTGTCTCTCTTCCCGAACTATGACTCCAGAACCTACCCTATCCGTACAGTGTCCCAACTTACCAGAGAAATCTATACTAACCCAGTTCTTGAGAACTTCGACGGTAGCTTCCGTGGTTCTGCCCAAGGTATCGAAGCTCCATCAGGAGCCCACACTTGATGGACATCTTGAACAGCATAACTATCTACACCGATGCTCACAGAGGAGAGTATTACTGGTCTGGACACCAGATCATGCCTCCTCCAGTTGATCAGCGGGCCCGAGTTTACCTTTCTCTCTATGACTATGGAACGCCGCTCCCACACGTATCGTGCTCAACTAGTCAG*  >MON87701_Cry-R: Cry1Ac followed by a transit peptide and pRbcS4 [GenBank: X68342, CP002684].  *GTTGT*CATGGCCTGCATGCAGTTGACGCGACCACCGGAATCGGTAAGGTCAGGAAGGTAAGAGAGAGTCTCAAACTTCTTCTTTCCAATCGGAGGCCACACCTGCATGCAGTTAACTCTTCCGCCGTTGCTTGTGATGGAAGTAATGTCGTTGTTAGCCTTGCGGGTGGCTGGGAAGGCAGCGGAGGACTTAAGTCCGTTGAAAGGAGCGACCATAGTGGCCTGAGCCGGAGAGGCAACCATAGTAGCGGAAGAGAGCATAGAGGAAGCCATTGTTCTTCTTTACTCTTTGTGTGACTGA**GGTTTGGTCTAGTGCTTTGGTCATCTATATATAATGATAACAACAATGAGAACAAGCTTTGGAGTGATCGGAGGGTCTAGGATACATGAGATTCAAGTGGACTAGGATCTACACCGTTGGATTTTGAGTGTGGATATGTGTGAGGTTAATTTTACTTGGTAACGGCCACAAAGGCCTAAGGAGAGGTGTTGAGACCCTTATCGGCTTGAACCGCTGGAATAATGCCACGTGGAAGATAATTCCATGAATCTTATCGTTATCTATGAGTGAAATTGTGTGATGGTGGGGTGGTGCTTGCTCATTTTACTTGCCTGGTGGGACTTGGCCCTTTCCTTATGGGGAATTTATATTTTACTTACTATAGAGCTTTCATACCTTTTTTTTTACCTTTGGATTTAGTTAATATATAATGGTATGATTCATGAATAAAAATGGGAAAATTTTTGAATTTTGTACTGCTTAAATTGCATAAGATTAAGGTGAAACTGGTGGAATTATATAATTTTTTTTCATTTTAAAAGCCAAAATTTGCCCTTTTTTACTAGAAATTTATAATATTTAGCAAAATTATTTTACCATTTCCAATTTAACATTGAAAATTAAGAATCTTTTTCAAAAAACAGAACATATGTTTATGTGTGAAGAATATATTCCCCCACTCAGTCTCTCTGTTACA**  >KeFeng-6_Cry-F: Cry1Ac [GenBank: KP195020]  *TCATCTGGGGTATCTTTGGTCCATCTCAATGGGATGCATTCCTGGTGCAAATTGAGCAGTTGATCAACCAGAGGATCGAAGAGTTCGCCAGGAACCAGGCCATCTCTAGGTTGGAAGGATTGAGCAATCTCTACCAAATCTATGCAGAGAGCTTCAGAGAGTGGGAAGCCGATCCTACTAACCCAGCTCTCCGCGAGGAAATGCGTATTCAATTCAACGACATGAACAGCGCCTTGACCACAGCTATCCCATTGTTCGCAGTCCAGAACTACCAAGTTCCTCTCTTGTCCGTGTACGTTCAAGCAGCTAATCTTCACCTCAGCGTGCTTCGAGACGTTAGCGTGTTTGGGCAAAGGTGGGGATTCGATGCTGCAACCATCAATAGCCGTTACAACGACCTTACTAGGCTGATTGGAAACTACACCGACCACGCTGTTCGTTGGTACAACACTGGCTTGGAGCGTGTCTGGGGTCCTGATTCTAGAGATTGGATTAGATACAACCAGTTCAGGAGAGAATTGACCCTCACAGTTTTGGACATTGTGTCTCTCTTCCCGAACTATGACTCCAGAACCTACCCTATCCGTACAGTGTCCCAACTTACCAGAGAAATCTATACTAACCCAGTTCTTGAGAACTTCGACGGTAGCTTCCGTGGTTCTGCCCAAGGTATCGAAGGCTCCATCAGGAGCCCACACTTGATGGACATCTTGAACAGCATAACTATCTACACCGATGCTCACAGAGGAGAGTATTACTGGTCTGGACACCAGATCATGGCCTCTCAGTGGATTCAGCGGGCCCGAGTTACCTTTCTCTTCTATGACTATGGGAAACGCGCTCACAACACGTATCGTGCTCAACTAGGTCAG*  >KeFeng-6_Cry-R: Cry1Ac followed by pUBI, CpTi and tNOS [GenBank: AM888351, HQ161056]  *TCCTCTAGAG*TCGACCTGCAGAAGTAACACCAAACAACAGGGTGAGCATCGACAAAAGAAACAGTACCAAGCAAATAAATAGCGTATGAAGGCAGGGCTAAAAAAATCCACATATAGCTGCTGCATATGCCATCATCCAAGTATATCAAGATCAAAATAATTA*T*CGCGCATTGCaaatcctgcatgtgtacacgatcaatgccaggcaagtgtcgttgccttgacgttgctgatttctgttacaaaccttgcaagtccagggatgaagatgatgagaaagatgaactctagatcaagctcgattctagagtcaa**gcagatcgttcaaacatttggcaataaagtttcttaagattgaatcctgttgccggtcttgcgatgattatcatataatttctgttgaattacgttaagcatgtaataattaacatgtaatgcatgacgttatttatgagatgggtttttatgattagagtcccgcaattatacatttaatacgcgatagaaaacaaaatatagcgcgcaaactaggataaattatcgcgcgcggtgtcatctatgttactagatc**GACCCTAGAGTCGAGGGGGGGCCCGGTACCCAACTTTTGTTCCCTTTAGTGAGGGTTAATTGCGCGCTTGGCGTAATCATGGTCATAGCTGTTTCCTGTGTGAATTGTTATCCGCTCACAATTCCACACGACATACGAGCCGGGAGCATAGGTGTAAAGCCTGGGGTGCCTAGTGACTGAGCTACTCACATTTATGCGTGCGCTCACTGCCGCTTTCAGTCGGCAACTGTCTGC  >MON15985_Cry-F: Cry1Ac [GenBank: KP195020]  *TCATCTGGGGTATCTTTGGTCCATCTCAATGGGATGCATTCCTGGTGCAAATTGAGCAGTTGATCAACCAGAGGATCGAAGAGTTCGCCAGGAACCAGGCCATCTCTAGGTTGGAAGGATTGAGCAATCTCTACCAAATCTATGCAGAGAGCTTCAGAGAGTGGGAAGCCGATCCTACTAACCCAGCTCTCCGCGAGGAAATGCGTATTCAATTCAACGACATGAACAGCGCCTTGACCACAGCTATCCCATTGTTCGCAGTCCAGAACTACCAAGTTCCTCTCTTGTCCGTGTACGTTCAAGCAGCTAATCTTCACCTCAGCGTGCTTCGAGACGTTAGCGTGTTTGGGCAAAGGTGGGGATTCGATGCTGCAACCATCAATAGCCGTTACAACGACCTTACTAGGCTGATTGGAAACTACACCGACCACGCTGTTCGTTGGTACAACACTGGCTTGGAGCGTGTCTGGGGTCCTGATTCTAGAGATTGGATTAGATACAACCAGTTCAGGAGAGAATTGACCCTCACAGTTTTGGACATTGTGTCTCTCTTCCCGAACTATGACTCCAGAACCTACCCTATCCGTACAGTGTCCCAACTTACCAGAGAAATCTATACTAACCCAGTTCTTGAGAACTTCGACGGTAGCTTCCGTGGTTCTGCCCAAGGTATCGAAGGCTCCATCAGGAGCCCACACTTGATGGACATCTTGAACAGCATAACTATCTACACCGATGCTCACAGAGGAGAGTATTACTGGTCTGGACACCAGATCATGGCCTCTCCAGTTGGATTCAGCGGGCCCGAGTTTACCTTTCTCTTCTATG*  >MON15985_Cry-R: Cry1Ac followed by p35S [GenBank: GU583853, KJ608138]  *TGTCATGGAGATCTGCTAGAGTCAGCTTGTCA*GCGTGTCCTCTCCAAATGAAATGAACTTCCTTATATAGAGGAAGGGTCTTGCGAAGGATAGTGGGATTGTGCGTCATCCCTTACGTCAGTGGAGATATCACATCAATCCACTTGCTTTGAAGACGTGGTTGGAACGTCTTCTTTTTCCACGATGCTCCTCGTGGGTGGGGGTCCATCTTTGGGACCACTGTCGGCAGAGGCATCTTCAACGATGGCCTTTCCTTTATCGCAATGATGGCATTTGTAGGAGCCACCTTCCTTTTCCACTATCTTCACAATAAAGTGACAGATAGCTGGGCAATGGAATCCGAGGAGGTTTCCGGATATTACCCTTTGTTGAAAAGTCTCAATCGGACCATCACATCAATCCACTTGCTTTGAAGACGTGGTTGGAACGTCTTCTTTTTCCACGATGCTCCTCGTGGGTGGGGGTCCATCTTTGGGACCACTGTCGGCAGAGGCATCTTCAACGATGGCCTTTCCTTTATCGCAATGATGGCATTTGTAGGAGCCACCTTCCTTTTCCACTATCTTCACAATAAAGTGACAGATAGCTGGGCAATGGAATCCGAGGAGGTTTCCGGATATTACCCTTTGTTGAAAAGTCTCAATCGGACCTGCAGCCAAGCTTGGCTGCAGGTCGACGGATCCCCGGGAGCACAGGATGACGCCCCGGAGGTAAACGACGCGGGTTCGGCCGTAATGGCCACTCCGGCGTGGATACCACGGCTTCCAGGGT  >281-24-236 x 3006-210-23_Cry-F: Cry1Ac [GenBank: U63372]  *ATCATTTGGGGATCTTTGGTCCCTCTCAATGGGACGCCTTTCTTGTACAGATAGAGCAGTCTAATTAACCAAAGAATAGAAGAATTCGCTAGGAACCAAGCCATCTCAAGGTTAGAAGGCCTCAGCAACCTTTACCAGATTTACGCAGAATCTTTTCGAGAGTGGGAAGCAGACCCGACCAATCCTGCCTTAAGAGAGGAGATGCGCATTCAATTCAATGACATGAACAGCGCGCTGACGACCGCAATTCCGCTCTTCGCCGTTCAGAATTACCAGGTTTCTC*  >281-24-236 x 3006-210-23_Cry-R: Cry1Ac followed by pUBI [GenBank: U63372, KY313416].  *TGGGTTTGTTGGTCCTTTTG*TTTGGATCCTCTAGAGTCGACCTGCAGAAGTAAGACCAAACAACAGGGTGAGCATCGACAAAAAGAAAACAGTACCAAGCAAATAAATAGCGTATGAAGGCAGGGCTAAAAAAATCCACATATAGCTGCTGCATATGCCATCATCCAAGTATATCAAGATCAAAATAATTATAAAACATACTTGTTTATTATAATAGATAGGTACTCAAGGTTAGAGCATATGAATAGATGCTGCATATGCCATCATGTATATGCATCAGTAAAACCCACATCAACATGTATACCTATCCTAGATCGATATTTCCATCCATCTTAAACTCGTAACTATGAAGATGTATGACACACACATACAGTTCCAAAATTAATAAATACACCAGGTAGTTTGAAACAGTATTCTACT  >MON89034_Cry-F: Cry2Ab2 [GenBank: KJ716235]  *TCATCTGGGCATCTTTGGCCCTCCCAGTGGGACGCCTTCCTGGTGCAAATCGAGCAGCTCATCAACCAGAGGATCGAGGAGTTCGCCAGGAACCAGGCCATCAGCCGCCTGGAGGGCCTCAGCAACCTCTACCAAATCTACGCTGAGGGCTTCCGCGAGTGGGAGGCCGACCCCACTAACCCAGCTCTCCGCGAGGAGATGCGCATCCAGTTCAACGACATGAACAGCGCCCTGACCACCGCCATCCCACTCTTCGCCGTCCAGAACTACCAAGTCCCGCTCCTGTCCGTGTACGTCCAGGCCGCCAACCTGCACCTCAGCGTGCTGAGGGACGTCAGCGTGTTTGG*  >MON89034_Cry-R: Cry2Ab2 followed by a transit peptide [GenBank: KJ716235, BT085253].  *TCAGGACGGA*GTTGTCCATGGCCTGCATGCAGCGGATGCGCCCGCCGGTCGACAGCGGCGGCAGGTACGACAGCGTCTCGAACTTCTTGTTGCCGTAGGCCGGCCACACCTGCACGTAGTACGTGTGTTTTGGTTCGTTTGGGGTTGGGAAATGGGATGGGATGGGCAACACACATCAGTCCATGCATGGATCATCAGTTCCCTTCCTA  >Bt176_Cry-F: Cry1Ab [GenBank: AY326434]  *ATCATCTGGGCATCTTCGGCCCAGCCAGTGGGACGCCTTCCTGGTGCAGATCGAGCAGCTGATCAACCAGCGCATCGAGGAGTTCGCCCGCAACCAGGCCATCAGCCGCCTGGAGGGCCTGAGCAACCTGTACCAAATCTACGCCGAGAGCTTCCGCGAGTGGGAGGCCGACCCCACCAACCCCGCCCTGCGCGAGGAGATGCGCATCCAGTTCAACGACATGAACAGCGCCCTGACCACCGCCATCCCCCTGTTCGCCGTGCAGAACTACCAGGGGC*  >Bt176_Cry-R: Cry1Ab followed by a part of the Zea mays calcium-dependent protein kinase gene [GenBank: AY326434, L27484].  *TTGTTGGATCC*ggctgcggcggggaacgagtgcggccgcctgctcccacggagggcagccagccacggacatgaacggcgagagatggaggggcgtatcgggaggaaaaaaaaaggtttctagattttgaagggtagggagggaatggcgggaaagaagagggcggcagggtgtctcgtgacagatatatataggctgcttgctgtttctggcaaaactgatcaacagcacttccagattgaagttgtg |
| --- |
