# Supplementary material for: Development and validation of an integrated DNA walking strategy to detect GMO expressing cry genes
Source: BMC Biotechnol. 2018 Jun 27;18:40. doi: 10.1186/s12896-018-0446-x (PMC6020286; doi:10.1186/s12896-018-0446-x)
Supplement: Supplementary file 4 — Alignement of the targeted Cry1Ab sequences from the T304–40 event sequenced from the PCR verification assay (1 and 2) with the reference sequence from the T304–40 event (reference) used for the design of the target-specific primers (surrounded by orange rectangles). (DOCX 506 kb) [file 12896_2018_446_MOESM4_ESM.docx]

**Additional file 4: Alignement of the targeted CryAb sequences from the T304-40 event sequenced from the PCR verification assay (1 and 2) with the reference sequence from the T304-40 event (reference) used for the design of the target-specific primers (surrounded by orange rectangles).**

| **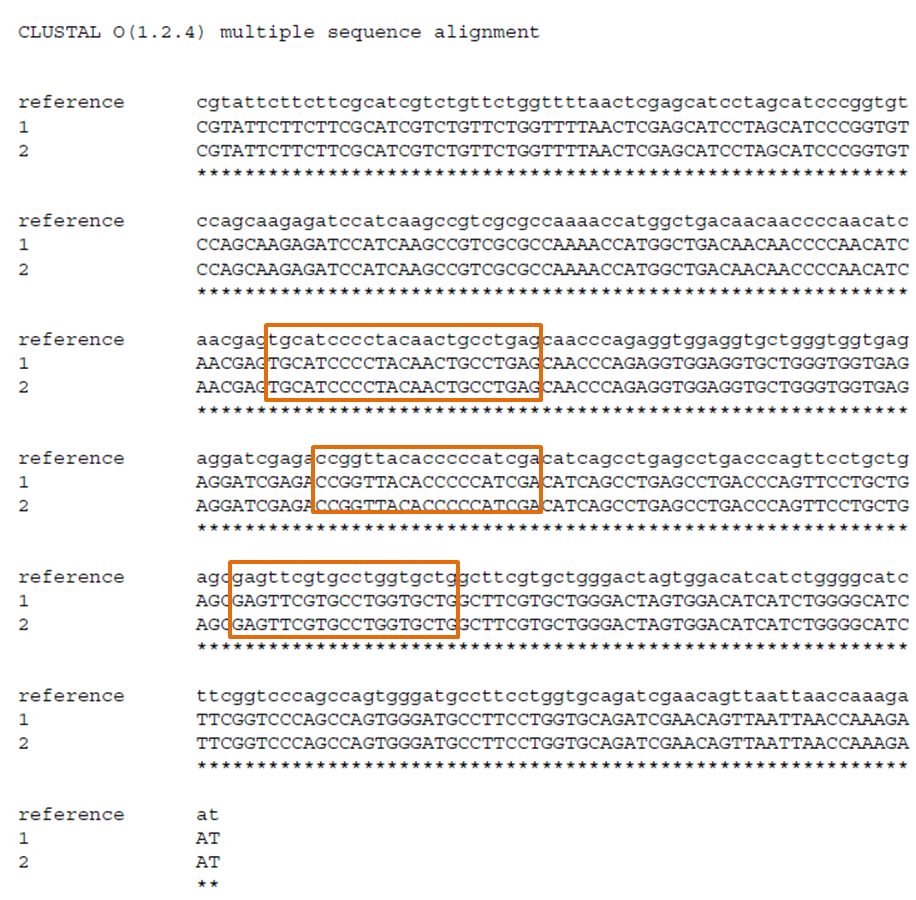** |
| --- |

In order to identify the potential reason(s) of the performance drop in term of sensitivity for the T304-40 event, a sequence from the T304-40 event including the targeted Cry1Ab region was amplified by PCR to be sequenced. As no nucleotide variation was observed between this amplicon sequence and the sequence from the T304-40 event used for the design of the target-specific primers, the observed performance drop could not be linked to the affinity of the target-specific primers (Additional file 3). Indeed, the few nucleotide variations observed with the T304-40 event are similar to the ones observed in the MON810 event, which is always detected as low as 20 HGE (Table 3, Additional file 1). Therefore, this performance drop could be related to a weaker affinity of the DRT primers used.
