# Supplementary material for: Development and validation of an integrated DNA walking strategy to detect GMO expressing cry genes
Source: BMC Biotechnol. 2018 Jun 27;18:40. doi: 10.1186/s12896-018-0446-x (PMC6020286; doi:10.1186/s12896-018-0446-x)
Supplement: Supplementary file 5 — Sequences of the sequenced amplicons from the processed food matrix (GeM MP17) coming from a GeMMA Scheme Proficiency Test. (DOCX 14 kb) [file 12896_2018_446_MOESM5_ESM.docx]

**Additional file 5: Sequences of the sequenced amplicons from the processed food matrix (GeM MP17) coming from a GeMMA Scheme Proficiency Test. Cry1Ab gene (italic); Intron hsp70 (underlined).**

| >GeM MP17_Cry-F  *CGTGACTCATCTGGGGCATCTTTGGCCCTCCCAGTGGGACGCCTTCCTGGTGCAAATCGAGCAGCTCATCAACCAGAGGATCGAGGAGTTCGCCAGGAACCAGGCCATCAGCCGCCTGGAGGGCCTCAGCAACCTCTACCAAATCTACGCTGAGAGCTTCCGCGAGTGGGAGGCCGACCCCACTAACCCAGCTCTCCGCGAGGAGATGCGCATCCAGTTCAACGACATGAACAGCGCCCTGACCACCGCCATCCCACTCTTCGCCGTCCAGAACTACCAAGTCCCGCTCCTGTCCGTGTACGTCCAGGCCGCCAACCTGCACCTCAGCGTGCTGAGGGACGTCAGCGTGTTTGGCCAGAGGTGGGGCTTCGACGCCGCCACCATCAACAGCCGCTACAACGACCTCACCAGGCTGATCGGCAACTACACCGACCACGCTGTCCGCTGGTACAACACTGGCCTGGAGCGCGTCTGGGGCCCTGATTCTAGAGACTGGATTCGCTACAACCAGTTCAGGCGCGAGCTGACCCTCACCGTCCTGGACATTGTGTCCCTCTTCCCGAACTACGACTCCCGCACCTACCCGATCCGCACCGTGTCCCAACTGACCCGCGAAATCTACACCAACCCCGTCCTGGAGAACTTCGACGGTAGCTTCAGGGGCAGCGCCCAGGGCATCGAGGGCTCCATCAGGAGCCCACACCTGATGGACATCCTCAACAGCATCACTATCTACACCGATGCCCACCGCGGCGAGTACTACTGGTCCGGCCACCAGATCATGACCTCCCCGTCGCTTCAGCGGCCCGAGTTTACCTTTCCTCACTACG*  >GeM MP17_Cry-R  TTGGGCTTCTGTCATGGCCGCTTGGTATCTGCATTACAATGAAATGAGCAAAGACTATGTGAGTAACACTGGTCAACACTAGGGAGAAGGCATCGAGCAAGATACGTATGTAAAGAGAAGCAATATAGTGTCAGTTGGTAGATACTA |
| --- |
