# Supplementary material for: Development and validation of an integrated DNA walking strategy to detect GMO expressing cry genes
Source: BMC Biotechnol. 2018 Jun 27;18:40. doi: 10.1186/s12896-018-0446-x (PMC6020286; doi:10.1186/s12896-018-0446-x)
Supplement: Supplementary file 6 — Sequences of oligonucleotides provided by the APAgeneTM GOLD Genome Walking Kit from BIO S&T. (DOCX 13 kb) [file 12896_2018_446_MOESM6_ESM.docx]

**Additional file 6: Sequences of oligonucleotides provided by the APAgeneTM GOLD Genome Walking Kit from BIO S&T.**

| **Oligonucleotide names** | **Oligonucleotide sequences** |
| --- | --- |
| DRT Primer A | 5’-GAACACGCGTCGTTTACCTCCXXXXGXXXXXTAGT-3’ |
| DRT Primer B | 5’-GAACACGCGTCGTTTACCTCCXXXXXXTAGT-3’ |
| DRT Primer C | 5’-GAACACGCGTCGTTTACCTCCXXXXXXTCAT-3’ |
| DRT Primer D | 5’-GAACACGCGTCGTTTACCTCCXXXXXGXXXXXCCTG-3’ |
| UAP-N1 | 5’-AGTCGGGAAGCAGTGGTATCAACGCAGAGTGGCCATTACGGCCGAACACGCGTCGTTTACCT-3’ |
| UAP-N2 | 5’- C CUG GAA GCA GTG GTA TCA ACG-3’ |
